# Relationship Between Salivary and Serum Cardiac Troponin I in Patients Undergoing Cardiac Surgery: A Prospective Longitudinal Observational Study

**DOI:** 10.3390/diagnostics16132077

**Published:** 2026-07-02

**Authors:** Ružica Mrkonjić, Andrej Šribar, Igor Rudež, Jadranka Ristić, Janko Bubnjar, Marin Pavlov, Anita Miljas, Željka Dujmić, Jasminka Peršec

**Affiliations:** 1 Department of Cardiovascular and Transplant Surgery, University Hospital Dubrava, 10000 Zagreb, Croatia; procelnik.kkir@kbd.hr; 2 Department of Anesthesiology, Resuscitation and Intensive Care Medicine, University Hospital Dubrava, 10000 Zagreb, Croatia; asribar@sfzg.hr (A.Š.); persec@sfzg.hr (J.P.); 3 School of Dental Medicine, University of Zagreb, 10000 Zagreb, Croatia; 4 School of Medicine, University of Zagreb, 10000 Zagreb, Croatia; 5 Department of Gynecology and Obstetrics, University Hospital Sveti Duh, 10000 Zagreb, Croatia; jadra.ristic@gmail.com; 6 Clinical Department of Laboratory Diagnostics, University Hospital Dubrava, 10000 Zagreb, Croatia; jbubnjar1@kbd.hr; 7 Department of Cardiology, University Hospital Dubrava, 10000 Zagreb, Croatia; marin.pavlov@gmail.com; 8 University North, 42000 Varaždin, Croatia; 9 General Hospital Dubrovnik, 20000 Dubrovnik, Croatia; anita.miljas1@gmail.com; 10 Department of Applied Ecology, Nursing and Maritime Studies, University of Dubrovnik, 20000 Dubrovnik, Croatia; 11 General Hospital “Dr. Josip Benčević”, 35000 Slavonski Brod, Croatia; dujmic.zeljka@gmail.com; 12 Faculty of Dental Medicine and Health Osijek, Josip Juraj Strossmayer University of Osijek, 31000 Osijek, Croatia

**Keywords:** cardiac troponin I, saliva, myocardial injury, biomarkers, salivary diagnostics

## Abstract

**Background/Objectives:** Cardiac troponin I (TnI) is the reference biomarker for detecting myocardial injury. Saliva has recently emerged as a potential non-invasive diagnostic fluid; however, evidence regarding the clinical utility of salivary TnI remains limited. This study aimed to compare serum and salivary TnI concentrations in patients undergoing cardiac surgery and to evaluate their relationship during the perioperative period. **Methods:** A prospective longitudinal observational study included 54 adult patients undergoing elective cardiac surgery with cardiopulmonary bypass and cardioplegic arrest. Serum and unstimulated saliva samples were collected 18–20 h before surgery, 18–20 h after surgery, and 42–44 h after surgery. TnI concentrations were measured using a high-sensitivity chemiluminescent immunoassay. Salivary pH, salivary flow rate, renal function, and fluid balance were also recorded. **Results:** Significant perioperative changes in TnI concentrations were observed in both serum and saliva (*p* < 0.001). Median salivary TnI increased from 3.0 ng/L preoperatively to 9.2 ng/L at 18–20 h postoperatively and decreased to 6.4 ng/L at 42–44 h. Median serum TnI increased from 10.2 ng/L to 2593.1 ng/L and subsequently decreased to 1204.5 ng/L. Despite similar temporal trends, no significant correlation was found between serum and salivary TnI concentrations at any time point. Ischemic time was positively associated with postoperative serum TnI concentrations (ρ = 0.347, *p* = 0.01), whereas no such association was observed for salivary TnI. Salivary TnI concentrations were not significantly associated with salivary flow rate or pH. **Conclusions:** Salivary TnI concentrations increased significantly following cardiac surgery, indicating measurable perioperative changes within the salivary compartment. However, no significant association was observed between salivary and serum TnI concentrations under the conditions investigated in this study. Therefore, the present findings do not support the use of salivary TnI as a surrogate marker of circulating troponin concentrations. Further analytical validation of high-sensitivity troponin assays in saliva and additional clinical studies are required before definitive conclusions regarding the biological significance and potential clinical utility of salivary troponin measurements can be made.

## 1. Introduction

Troponin I (TnI) is one of the three components of the troponin complex that regulates striated muscle contraction. Together with troponin C (TnC), which binds calcium and initiates the contraction process, and troponin T (TnT), which anchors the complex to tropomyosin, it participates in the control of actin–myosin interactions. TnI acts as the inhibitory subunit by blocking myosin-binding sites on actin.

TnC is present in all skeletal muscles, whereas TnI and TnT are highly specific to cardiac muscle tissue due to differences in amino acid sequence and expression patterns. TnI is considered more specific for cardiac muscle tissue because TnT can be re-expressed in regenerating skeletal muscle in certain neuromuscular diseases and is also frequently elevated in patients with impaired renal function in the absence of myocardial injury. Therefore, TnI is considered a more reliable indicator of acute myocardial injury [1]. Following myocardial injury caused by ischemia, trauma, or inflammation, TnI is released into the circulation. Increased blood concentrations may indicate cardiomyocyte necrosis but may also reflect reversible processes such as increased membrane permeability, apoptosis, or oxidative stress [1].

Due to its cardiac specificity and release into the circulation following cardiomyocyte injury, TnI is currently considered one of the most important biomarkers of myocardial injury. Its application in clinical practice has significantly improved the diagnosis of acute myocardial infarction and has gradually replaced previously used biomarkers such as creatine kinase-MB (CK-MB). Further advances in laboratory diagnostics have led to the development of high-sensitivity assays capable of measuring very low troponin concentrations in blood.

High-sensitivity (hs) assays have made TnI a key component in the diagnosis of acute myocardial ischemia. They can detect very low concentrations, enabling early recognition of even minor myocardial injury, sometimes within only a few hours after symptom onset [2]. Results are interpreted according to the 99th percentile upper reference limit, with the exact value depending on the assay used and population characteristics, particularly age and sex [2,3]. Evidence suggests that reference values for hs-troponin may differ among ethnic groups, although this effect is generally smaller and less consistent than differences associated with sex and age. In certain clinical situations, such as the diagnosis of perioperative myocardial infarction (Type 5 MI) in patients following cardiac surgery, specific guideline-defined criteria are applied [4].

Troponin is traditionally measured from venous blood, which requires venipuncture and trained personnel. In an effort to simplify the diagnosis of cardiac injury, the potential of salivary diagnostics as a non-invasive alternative has recently been investigated. Saliva contains numerous biomolecules, sample collection is simple, and serial measurements are easier to perform, particularly in patients in whom blood sampling is difficult.

Saliva contains numerous proteins, hormones, nucleic acids and other biomarkers that may reflect both oral and systemic diseases [5]. Amid the growing interest in salivary biomarkers, several studies have investigated the possibility of measuring TnI in saliva. Some authors, such as Mirzaii et al. [5] and Mishra et al. [6], found a strong correlation between salivary and serum TnI concentrations. Others reported only moderate correlations or considerable variability [7,8].

The pre-analytical phase is particularly sensitive in saliva analysis. Sample quality is influenced by the method and timing of collection, oral hygiene, nutritional status, contamination (blood, food debris, mucus), as well as transport and storage conditions [9,10,11,12,13]. It is likely that some of the inconsistency observed in previous studies results from the fact that these studies were conducted in patients with acute myocardial infarction, in whom pre-analytical conditions are difficult to control because of the urgency of care and clinical instability.

Cardiac surgical patients represent an excellent model for investigating salivary TnI because they experience predictable and temporally well-defined myocardial injury. In this population, elevated serum troponin concentrations are common even in the absence of infarction; therefore, specific diagnostic thresholds are used in the assessment of perioperative myocardial infarction. According to current ESC guidelines, Type 5 MI is defined as an increase in TnI or TnT to more than ten times the 99th percentile upper reference limit within 48 h after surgery, together with at least one additional criterion: new pathological Q waves, new left bundle branch block, angiographic evidence of graft or coronary artery occlusion, or imaging evidence of new regional myocardial injury [3,14].

To date, no study has been conducted to evaluate the diagnostic or prognostic value of salivary TnI in cardiac surgical patients using high-sensitivity testing methods. Because these patients remain in a strictly controlled hospital environment throughout the perioperative period, allowing standardized saliva collection under controlled conditions of diet, physical activity, and oral hygiene, this population provides a unique opportunity to assess the reliability of salivary diagnostics.

The primary objective of this study was to evaluate the association between salivary and serum TnI concentrations in patients undergoing elective cardiac surgery with cardiopulmonary bypass and cardioplegic arrest. Secondary objectives were to assess perioperative changes in salivary TnI concentrations and to evaluate the potential influence of salivary pH, salivary flow rate, renal function, and hydration status on salivary troponin measurements.

## 2. Materials and Methods

### 2.1. Study Design and Participants

This prospective longitudinal observational study was conducted at the Department of Cardiac and Transplant Surgery and the Department of Anesthesiology, Reanimatology and Intensive Care Medicine, University Hospital Dubrava, Zagreb, Croatia.

The study included 54 adult patients undergoing elective cardiac surgery with cardiopulmonary bypass and cardioplegic arrest. Surgical procedures included coronary artery bypass grafting (CABG), aortic valve replacement or repair (AVR), mitral valve replacement or repair (MVR), and combined procedures. Cardiopulmonary bypass and cardioplegic arrest were used in all patients, resulting in controlled perioperative myocardial ischemia and a predictable postoperative increase in TnI concentrations.

Exclusion criteria included active malignant disease, salivary gland disorders, severe xerostomia, active oral infection, maxillofacial conditions affecting saliva production, severe chronic kidney disease (estimated glomerular filtration rate (eGFR) < 30 mL/min/1.73 m^2^), inability to provide an adequate saliva sample, administration of medications known to significantly affect salivary secretion within 48 h before sampling, and refusal to provide written informed consent.

#### Sample Size Calculation

The primary objective of this study was to evaluate the association between salivary and serum TI concentrations. Therefore, the sample size calculation was based on correlation analysis. Assuming a clinically meaningful moderate correlation coefficient of r = 0.50, a two-sided significance level of α = 0.05, and a statistical power of 80% (1 − β = 0.80), a minimum of 26 participants was required. Sample size calculations were performed using G*Power software (version 3.1, Heinrich Heine University Düsseldorf, Germany).

To account for potential exclusions due to insufficient saliva volume, sample contamination, missing measurements, or participant withdrawal, the target sample size was increased to approximately 50 participants. A total of 54 patients were ultimately included, exceeding the minimum sample size requirement for the primary correlation analysis by more than twofold and providing adequate statistical power to detect clinically meaningful moderate associations between salivary and serum TnI concentrations.

### 2.2. Pilot Study and Selection of the Saliva Collection Method

Prior to the main study, a pilot investigation was conducted to evaluate the feasibility and reproducibility of saliva collection in cardiac surgical patients. Initially, unstimulated saliva was collected by direct expectoration into a collection tube (spitting method), which is considered the reference (“gold standard”) method for unstimulated saliva sampling [9]. However, difficulties in obtaining adequate saliva volume and increased variability of measured salivary TnI concentrations were observed, particularly during the postoperative period.

Given the practical challenges associated with direct expectoration in postoperative cardiac surgical patients, a standardized absorbent swab-based collection method (SalivaBio Oral Swab, SOS) devices (Salimetrics LLC, State College, PA, USA) was selected for further evaluation.

Comparison of unstimulated saliva collected by direct expectoration and SOSdevices was performed in 10 participants. Salivary TnI concentrations obtained by the two methods demonstrated a strong correlation (r = 0.81). However, measurements obtained using the expectoration method showed greater variability (SD = 12.4 ng/L) compared with SOS sampling (SD = 7.5 ng/L). Bland–Altman analysis demonstrated a mean difference of 3.2 ng/L with limits of agreement ranging from −7.6 to 14.0 ng/L.

Based on the pilot study findings, SOS sampling demonstrated superior practicality and reproducibility under clinical conditions and was therefore selected for use in the study.

### 2.3. Saliva and Blood Sampling

Unstimulated whole saliva was collected using SOS devices. Saliva and venous blood samples were obtained simultaneously at three predefined time points: 18–20 h before surgery (baseline), 18–20 h after surgery, and 42–44 h after surgery.

To minimize circadian variability, all saliva samples were collected between 09:00 and 10:00 a.m. Participants were instructed to refrain from eating, drinking beverages other than water, smoking, and oral hygiene procedures for at least 60 min before sample collection. Water intake was permitted up to 30 min before sampling.

Prior to collection, the oral cavity was visually inspected for visible blood contamination, ulcerations, and signs of active oral disease. The SOS swab was placed in the sublingual region for 2 min (±10 sec) without chewing or active suction.

Immediately after collection, salivary pH was measured using Hydrion S/R indicator strips (Micro Essential Laboratory, Brooklyn, NY, USA). Salivary flow rate was determined gravimetrically by weighing the collection device before and after sample collection. The difference in weight was converted to saliva volume assuming a saliva density of 1 g/mL and expressed as mL/min according to the sampling duration.

Samples were transported to the laboratory at 2–8 °C and centrifuged at 3500 rpm for 10 min. Following centrifugation, saliva samples were visually assessed for volume, clarity, and discoloration. Samples with visible blood contamination or insufficient sample volume were excluded from analysis. Processed samples were stored at −80 °C until laboratory analysis.

### 2.4. Troponin I Determination

TnI concentrations in serum and saliva were measured using the Access High-Sensitivity Troponin I assay (Access hsTnI; Beckman Coulter, Brea, CA, USA) on the Access 2 Immunoassay Analyzer. The assay is based on chemiluminescent immunoassay (CLIA) technology and has a reported limit of detection of 2.3 ng/L according to the manufacturer’s specifications [15,16].

Although validated for serum analysis, the assay was applied to saliva because of its high analytical sensitivity and ability to detect low troponin concentrations. The assay has not been formally validated for salivary measurements, and matrix-related interference associated with the salivary environment cannot be excluded. Commercially available ELISA assays were not used for comparative validation because their limits of detection exceeded the expected concentration range of salivary TnI and were therefore unsuitable for reliable comparison.

#### Preliminary Analytical Verification of the Assay in Saliva

Because the Access High-Sensitivity Troponin I assay (Access hsTnI; Beckman Coulter, Brea, CA, USA) is validated for serum but not for saliva, a preliminary analytical verification was performed prior to the main study to assess the suitability of the assay for salivary measurements.

Repeatability was assessed using two saliva samples analyzed ten consecutive times under identical conditions.

Dilutional linearity was evaluated using serial mixtures of saliva samples with high and low troponin I concentrations prepared at predefined ratios (100:0, 75:25, 50:50, 25:75, and 0:100).

The objective of this verification was not to perform a complete analytical validation of the assay for salivary use, but rather to assess its basic analytical performance characteristics before application in this exploratory clinical study.

### 2.5. Clinical and Laboratory Variables

Demographic characteristics including age, sex, body weight, height, and body mass index (BMI) were recorded for all participants. Clinical variables included type of surgical procedure, duration of cardiopulmonary bypass, aortic cross-clamp time, myocardial ischemia duration, perioperative fluid balance, and relevant comorbidities.

Routine laboratory parameters included hemoglobin concentration, leukocyte count, serum creatinine concentration, estimated glomerular filtration rate (eGFR), and C-reactive protein (CRP). Salivary variables included pH and salivary flow rate.

Clinical and laboratory data were obtained from the hospital information system and medical records of University Hospital Dubrava.

### 2.6. Statistical Analysis

Categorical variables are presented as absolute frequencies and percentages. Continuous variables were tested for normality using the Shapiro–Wilk test and are presented as median and interquartile range (IQR) or mean ± standard deviation (SD), as appropriate.

Differences between two independent groups were analyzed using the Mann–Whitney U test with Hodges–Lehmann estimates and corresponding 95% confidence intervals. Comparisons among three or more independent groups were performed using the Kruskal–Wallis test with Conover post hoc correction. Repeated measurements were analyzed using Friedman’s test with post hoc Conover comparisons.

The primary analysis of the association between salivary and serum TnI concentrations across repeated measurements was performed using a linear mixed-effects model. Associations between continuous variables at individual time points were additionally assessed using Spearman’s rank correlation coefficient (ρ). Agreement between salivary TnI concentrations obtained using direct expectoration and SOS sampling in the pilot study was assessed using Bland–Altman analysis. The relationship between salivary pH and salivary TnI concentrations was additionally evaluated using bivariate linear regression analysis.

Variables with non-normal distribution, including serum and salivary TnI concentrations, were logarithmically transformed (natural logarithm, ln) before linear regression and mixed-effects analyses.

Multivariable linear regression models were used to assess whether salivary TnI independently predicted serum TnI concentrations after adjustment for age, sex, myocardial ischemia duration, and fluid balance.

Covariates were selected a priori based on biological plausibility and previously reported factors known to influence perioperative troponin concentrations. To reduce the risk of model overfitting given the modest sample size, the number of covariates included in the multivariable models was intentionally limited.

To evaluate the association between salivary and serum TnI concentrations across repeated measurements, a linear mixed-effects model was constructed with ln-transformed serum TnI concentration as the dependent variable. Ln-transformed salivary TnI concentration and time point were included as fixed effects, while participant was included as a random effect to account for within-subject correlation arising from repeated measurements. A compound symmetry covariance structure was specified for repeated measurements. Results are presented as regression coefficients (β) with corresponding 95% confidence intervals (95% CI). Model fit was assessed using the −2 Restricted Log Likelihood, Akaike Information Criterion (AIC), and Bayesian Information Criterion (BIC).

Because several secondary and exploratory analyses were performed, the corresponding *p*-values should be interpreted as exploratory and hypothesis-generating. Formal adjustment for multiple testing was not applied because the primary analyses were predefined and the secondary analyses were intended to explore potential factors influencing salivary TnI concentrations.

Statistical analyses were performed using MedCalc Statistical Software version 23.5.2 (MedCalc Software Ltd., Ostend, Belgium). A two-sided *p*-value < 0.05 was considered statistically significant.

### 2.7. Ethical Considerations

The study was conducted in accordance with the Declaration of Helsinki and approved by the Ethics Committee of University Hospital Dubrava, Zagreb, Croatia (Approval No. 2024/0206-06), and by the Ethics Committee of the School of Dental Medicine, University of Zagreb, Croatia (Approval No. 05-PA-5-28/2024). Following modification of the saliva collection protocol from the spitting method to SalivaBio Oral Swab collection, additional approval was obtained from the Ethics Committee of the School of Dental Medicine, University of Zagreb (Approval No. 251-60-4/119-3). Written informed consent was obtained from all participants prior to enrolment in the study.

## 3. Results

### 3.1. Participant Characteristics

A total of 54 patients undergoing elective cardiac surgery with cardiopulmonary bypass were included in the study. The majority of participants were men (45/54, 83.3%), and the median age was 65 years (IQR 53.75–72.00). Most patients were younger than 70 years (70.4%). The median body mass index was 28.69 kg/m^2^ (IQR 26.28–30.72), indicating that most participants were overweight or obese (Table 1).

Comorbidities were present in 44 patients (81.5%). The most common comorbidities were arterial hypertension (74.1%), dyslipidemia (64.8%), obesity (32.0%), and diabetes mellitus (29.6%). The most frequently performed surgical procedures were coronary artery bypass grafting (CABG; 42.6%) and aortic valve replacement (AVR; 40.7%). The median cardiopulmonary bypass time was 76 min (IQR 64–93), while the median myocardial ischemia time was 50 min (IQR 37–61).

### 3.2. Results of the Preliminary Analytical Verification of the Assay

Repeatability testing demonstrated coefficients of variation of 3.1% and 4.5% for the high- and low-concentration saliva samples, respectively. Dilutional linearity analysis demonstrated a strong linear relationship across the investigated concentration range (r = 0.99; R^2^ = 0.98). The results are summarized in Table 2.

### 3.3. Perioperative Laboratory Parameters, Hydration Status, and Salivary Flow Rate

Significant perioperative changes were observed in all evaluated laboratory parameters. Glomerular filtration rate (GFR) reached its lowest values during the early postoperative period and subsequently recovered at the final measurement (*p* = 0.02). In contrast, inflammatory markers increased markedly after surgery. Median C-reactive protein (CRP) concentrations increased from 1.7 mg/L preoperatively to 48.7 mg/L at 18–20 h and 115.2 mg/L at 42–44 h postoperatively (*p* < 0.001). A similar pattern was observed for leukocyte counts, which increased from 6.8 × 10^9^/L preoperatively to 11.4 × 10^9^/L and 10.9 × 10^9^/L at the respective postoperative measurements (*p* < 0.001). Hemoglobin concentrations decreased significantly throughout the observation period, from a median of 132 g/L before surgery to 106 g/L at 42–44 h postoperatively (*p* < 0.001).

Postoperative fluid balance demonstrated substantial interindividual variability. Median cumulative fluid balance was −490 mL (IQR −1610 to 435 mL). Salivary flow rate decreased markedly in the early postoperative period and partially recovered by 42–44 h after surgery. Transfusion therapy was common, with 37 patients (68.5%) receiving packed red blood cells and 17 patients (31.5%) receiving fresh frozen plasma (Table 3).

### 3.4. Perioperative Changes in Serum and Salivary Troponin I Concentrations

Significant perioperative changes were observed in both serum and salivary TnI concentrations (Friedman test, *p* < 0.001 for both). Median salivary TnI increased from 3.0 ng/L (IQR 2.3–4.7) before surgery to 9.2 ng/L (IQR 4.8–17.8) at 18–20 h postoperatively, followed by a decrease to 6.4 ng/L (IQR 3.4–13.8) at 42–44 h after surgery (*p* < 0.001). Median salivary TnI concentrations increased by approximately 139% during the early postoperative period compared with baseline. A similar temporal pattern was observed for serum TnI, with concentrations increasing from 10.2 ng/L (IQR 5.1–19.5) preoperatively to 2593.1 ng/L (IQR 1682.9–4775.6) at 18–20 h postoperatively and subsequently decreasing to 1204.5 ng/L (IQR 687.4–2306.3) at the final measurement (*p* < 0.001). The highest concentrations of both biomarkers were recorded during the first postoperative measurement, indicating a pronounced biomarker response to myocardial injury associated with cardiac surgery (Table 4).

Despite these parallel postoperative increases, no significant correlations were observed between serum and salivary TnI concentrations at any measurement point (all *p* > 0.05).

Individual patient trajectories of salivary TnI concentrations across the three study time points are presented in Supplementary Figure S1, illustrating the substantial inter-individual variability underlying the observed group-level trends. Notably, some participants demonstrated decreases in salivary TnI concentrations despite the overall postoperative increase observed in the study population.

Furthermore, neither age nor sex significantly influenced serum or salivary TnI concentrations during the study period. A significant positive association was identified between myocardial ischemia time and serum TnI concentrations measured 18–20 h after surgery (ρ = 0.347, *p* = 0.01), whereas no such association was observed for salivary TnI.

**Figure 1:**
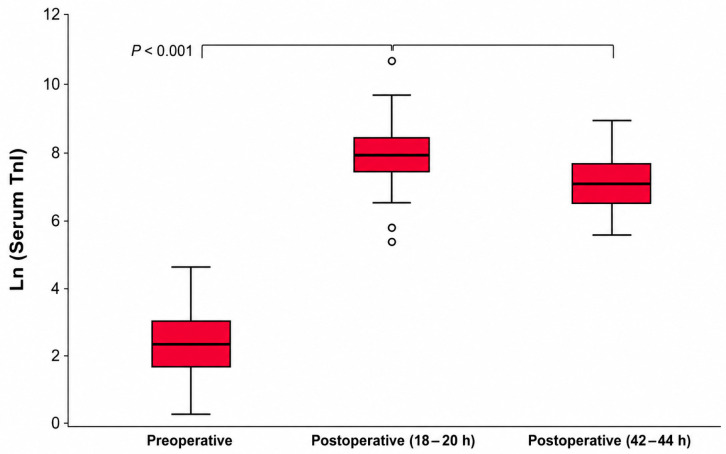
Box-plot presentation of ln-transformed serum troponin I concentrations during the perioperative period. The box represents the median and interquartile range, whereas whiskers indicate the range of values excluding outliers.

**Figure 2:**
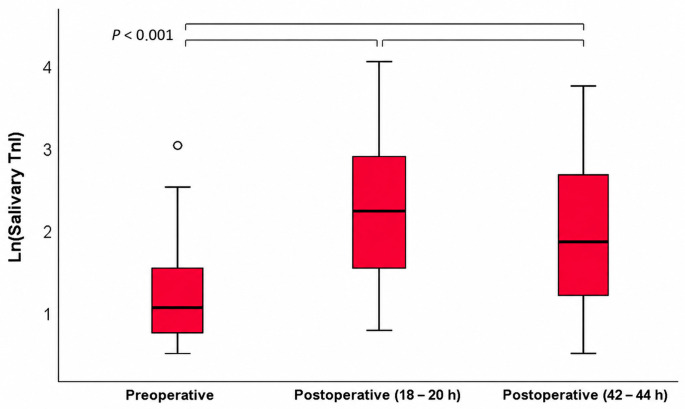
Box-plot presentation of ln-transformed salivary troponin I concentrations during the perioperative period. The box represents the median and interquartile range, whereas whiskers indicate the range of values excluding outliers.

No significant differences in serum or salivary troponin I concentrations were observed according to sex or age group at any study time point (all *p* > 0.05). The perioperative changes in serum TnI concentrations are illustrated in Figure 1. The corresponding perioperative changes in salivary TnI concentrations are shown in Figure 2.

**Table 4:** Perioperative Changes in Serum and Salivary Troponin I Concentrations and Subgroup Analyses.

Variable	Preoperative	Postoperative (18–20 h)	Postoperative (42–44 h)	*p*-Value
OVERALL POPULATION (*n* = 54)				
Salivary TnI (ng/L)	3.0 (2.3–4.7)	9.2 (4.8–17.8)	6.4 (3.4–13.8)	<0.001
Serum TnI (ng/L)	10.2 (5.1–19.5)	2593.1 (1682.9–4775.6)	1204.5 (687.4–2306.3)	<0.001
SEX SUBGROUP ANALYSIS				
Salivary TnI–males (*n* = 45)	2.8 (2.3–4.7)	8.5 (4.5–16.8)	6.6 (3.3–13.9)	
Salivary TnI–females (*n* = 9)	3.7 (2.3–4.9)	11.4 (8.9–34.4)	5.3 (3.6–13.6)	
Between-group *p*-value	0.46	0.11	0.89	
Serum TnI–males (*n* = 45)	11.3 (5.3–19.3)	2521.3 (1729.0–4230.5)	1254.5 (791.6–2302.6)	
Serum TnI–females (*n* = 9)	6.7 (4.3–20.8)	1875.0 (1637.1–5749.0)	848.6 (614.9–2525.7)	
Between-group *p*-value	0.69	0.78	0.31	
AGE SUBGROUP ANALYSIS				
Salivary TnI–<70 years (*n* = 38)	2.8 (2.3–4.8)	9.2 (4.3–16.6)	6.1 (3.4–11.8)	
Salivary TnI–≥70 years (*n* = 16)	3.7 (2.3–4.4)	10.0 (5.9–26.9)	8.4 (4.9–22.2)	
Between-group *p*-value	0.46	0.38	0.15	
Serum TnI–<70 years (*n* = 38)	10.7 (5.1–19.4)	2664.9 (1707.3–4409.6)	1137.0 (649.0–2074.2)	
Serum TnI–≥70 years (*n* = 16)	9.5 (3.7–19.5)	1875.0 (1558.2–4897.9)	1217.0 (801.1–4101.9)	
Between-group *p*-value	0.84	0.27	0.54	

Data are presented as median (interquartile range). Overall comparisons were performed using Friedman repeated-measures analysis with Conover post hoc testing. Between-group comparisons were performed using the Mann–Whitney U test. Abbreviation: TnI, troponin I.

Sensitivity analyses were additionally performed according to surgical category. Procedures were grouped as isolated coronary artery bypass grafting (CABG; *n* = 23), isolated valve surgery (aortic or mitral valve surgery; *n* = 27), and combined or complex procedures (*n* = 4). Correlation analyses between salivary and serum TnI concentrations were repeated within each subgroup. No statistically significant associations were observed within either the CABG group (Spearman ρ ranging from −0.22 to −0.09, all *p* > 0.30) or the valve surgery group (Spearman ρ ranging from −0.18 to 0.24, all *p* > 0.25) at any study time point. Because of the small number of patients undergoing combined or complex procedures, subgroup analyses in this category were considered exploratory. Overall, stratification by surgical category did not materially alter the principal findings of the study.

### 3.5. Absolute and Relative Changes in Serum and Salivary Troponin I Concentrations

The largest absolute and relative changes were observed for serum TnI during the early postoperative period. Median absolute serum TnI increased by 2507.8 ng/L at 18–20 h and by 1159.3 ng/L at 42–44 h after surgery. In contrast, salivary TnI showed substantially smaller increases, with median absolute changes of 4.75 ng/L and 3.55 ng/L at the respective postoperative measurements. A wide range of absolute and relative changes was observed, particularly for serum TnI, indicating considerable interindividual variability in the biomarker response to surgery.

Subgroup analyses revealed no significant differences in absolute or relative changes according to sex or age group. Likewise, no significant correlations were observed between changes in serum and salivary TnI concentrations. The only significant association identified was a positive correlation between ischemia time and the absolute increase in serum TnI measured 18–20 h after surgery (ρ = 0.351, *p* = 0.01), indicating a greater postoperative serum troponin response in patients exposed to longer myocardial ischemia. Among the evaluated perioperative variables, myocardial ischemia duration was the only factor significantly associated with postoperative serum TnI concentrations. No significant association was observed between myocardial ischemia duration and salivary TnI concentrations (Table 5).

### 3.6. Association Between Salivary pH and Salivary Troponin I

Salivary pH changed significantly during the perioperative period, decreasing from a median value of 7.0 preoperatively to 6.1 at 18–20 h postoperatively and partially recovering to 6.4 at 42–44 h after surgery (*p* < 0.001). Despite these changes, no significant correlations were observed between salivary pH and salivary TnI concentrations at any measurement point. Similarly, neither absolute changes in salivary troponin I nor ln-transformed salivary TnI concentrations showed significant associations with salivary pH in correlation or regression analyses.

However, subgroup analysis demonstrated that patients with salivary pH values below 6.0 at 42–44 h postoperatively had significantly higher salivary TnI concentrations and greater absolute increases in salivary TnI compared with patients with pH ≥ 6.0 (both *p* = 0.02). These findings suggest that although salivary pH is not a major determinant of salivary TnI concentrations overall, markedly acidic saliva may be associated with higher postoperative salivary TnI levels (Table 6).

### 3.7. Influence of Renal Function, Hydration Status and Salivary Flow Rate on Troponin I Concentrations

To evaluate the potential influence of renal function, hydration status, and salivary flow on TnI concentrations, analyses were performed examining associations between glomerular filtration rate (GFR), fluid balance, salivary flow rate, and both serum and salivary TnI concentrations throughout the perioperative period. Significant correlations were observed between preoperative GFR and TnI concentrations, with higher GFR values associated with higher salivary TnI concentrations and lower serum TnI concentrations. In contrast, postoperative GFR values showed limited associations with TnI concentrations, and no significant relationships were identified between GFR and perioperative changes in TnI levels.

Hydration status demonstrated a modest effect on serum TnI. Greater positive fluid balance was associated with higher postoperative serum TnI concentrations and greater absolute increases in serum TnI, whereas no significant associations were observed for salivary TnI concentrations or their perioperative changes. Similarly, salivary flow rate was not significantly associated with salivary TnI concentrations or with absolute and relative changes in salivary TnI. A summary of the associations between renal function, hydration status, salivary flow rate, and TnI concentrations is presented in Table 7.

Additional sensitivity analyses were performed according to preoperative renal function. Patients were stratified into two groups based on preoperative GFR (<60 and ≥60 mL/min/1.73 m^2^). Correlation analyses between salivary and serum troponin I concentrations were repeated within each subgroup. No statistically significant associations were observed at any study time point in either renal function category. Although a moderate inverse correlation was observed preoperatively in patients with GFR < 60 mL/min/1.73 m^2^ (ρ = −0.623, *p* = 0.054), this finding did not reach statistical significance and was based on a small number of participants. Overall, stratification by renal function did not materially alter the principal findings of the study.

### 3.8. Association of Independent Factors with Serum Troponin I Concentrations During the Perioperative Period

Due to the fact that the majority of patients exhibited postoperative serum TnI concentrations exceeding 10 times the 99th percentile upper reference limit, only a small number of participants did not meet the criteria for perioperative myocardial injury. Consequently, the highly unbalanced distribution of outcomes precluded a meaningful assessment of diagnostic accuracy using ROC curve analysis.

To investigate whether salivary TnI could serve as an independent marker of perioperative myocardial injury, multivariable linear regression analyses were performed using ln-transformed serum and salivary TnI concentrations. The models were adjusted for age, sex, ischemic time, and fluid balance. Neither postoperative model demonstrated a significant independent association between salivary and serum TnI concentrations. In the 24-h postoperative model, fluid balance during the first 24 h was the only significant independent predictor of serum TnI concentration. In contrast, no significant predictors were identified in the 48-h postoperative model. A summary of the multivariable regression analyses is presented in Table 8.

### 3.9. Association Between Salivary and Serum Troponin I During the Perioperative Period

To evaluate the longitudinal relationship between salivary and serum TnI concentrations throughout the perioperative period, a linear mixed-effects model was performed using natural logarithm (ln)-transformed TnI concentrations. The model included a random intercept for each participant to account for repeated measurements (54 patients; 156 observations in total).

No significant association was observed between ln-transformed salivary and serum TnI concentrations. In contrast, time had a significant effect on serum TnI concentrations. Compared with preoperative values, serum TnI concentrations were significantly higher at both postoperative time points (18–20 h and 42–44 h after surgery). Model fit indices were as follows: −2 Restricted Log Likelihood = 411.953, Akaike Information Criterion (AIC) = 417.953, and Bayesian Information Criterion (BIC) = 426.985.

A summary of the linear mixed-effects model is presented in Table 9.

## 4. Discussion

### 4.1. Principal Findings

The present study evaluated perioperative changes in salivary and serum cardiac troponin I concentrations in patients undergoing cardiac surgery with cardiopulmonary bypass and cardioplegic arrest. Cardiac troponins are structural proteins of the contractile apparatus of cardiomyocytes and play a fundamental role in myocardial contraction [1]. Due to their high cardiac specificity and sensitivity, cardiac troponins have become the cornerstone biomarkers for the detection of myocardial injury and are central to the Fourth Universal Definition of Myocardial Infarction [1,2].

The principal findings of the present study were: (1) both serum and salivary TnI concentrations increased significantly following surgery; (2) despite similar temporal trends, salivary TnI concentrations did not correlate significantly with serum TnI concentrations; (3) salivary TnI concentrations were not substantially influenced by salivary flow rate and demonstrated only limited associations with salivary pH; and (4) renal function and hydration status exerted a greater influence on serum than salivary troponin concentrations.

Cardiac surgery provides a unique model of controlled myocardial injury because the timing and duration of ischemia are known and occur under highly standardized conditions. The marked postoperative increase in serum TnI observed in this study was therefore expected and reflects procedure-related myocardial injury associated with cardioplegic arrest, cardiopulmonary bypass, and surgical manipulation of the heart [14]. Consistent with previous reports, most patients exceeded the threshold commonly used to define perioperative myocardial injury after cardiac surgery [2,14].

Importantly, despite the parallel postoperative increases observed in both biological matrices, no significant association was identified between salivary and serum TnI concentrations at any study time point. Because the final sample included 54 participants, exceeding the a priori requirement of 26 participants for detection of a moderate correlation (r = 0.50), the absence of a significant association is unlikely to be explained solely by insufficient statistical power.

Collectively, these findings indicate that salivary TnI concentrations increase significantly following cardiac surgery-induced myocardial injury but do not exhibit a clinically meaningful quantitative relationship with serum TnI concentrations.

### 4.2. Comparison with Previous Studies

Saliva has emerged as an attractive diagnostic fluid because it can be collected non-invasively, repeatedly, and without the need for specialized personnel [10,17,18]. Advances in salivary diagnostics have demonstrated that saliva contains numerous proteins, hormones, antibodies, nucleic acids, metabolites, and inflammatory mediators that may reflect both oral and systemic physiological processes [10,11,17,18,19,20]. Consequently, saliva has attracted increasing interest as a potential medium for cardiovascular biomarker testing [7,8,15].

In the present study, salivary TnI concentrations increased significantly after surgery, demonstrating that myocardial injury is accompanied by measurable changes in salivary troponin concentrations. These findings support the observations of Mirzaii-Dizgah et al. [5] who first reported elevated salivary TnI concentrations in patients with acute myocardial infarction, and Mishra et al. [6], who suggested that salivary TnI may represent a novel biomarker of myocardial injury. Furthermore, the present findings are consistent with systematic reviews conducted by Tuttolomondo et al. [21] and Meleti et al. [4] which identified saliva as a promising medium for systemic disease biomarkers and highlighted the growing interest in salivary cardiac troponins. Chaulin and Duplyakov [8] similarly reported detectable salivary cardiac troponin concentrations in patients with acute myocardial infarction, although substantial variability among studies was noted.

Despite the significant postoperative increase in both biological matrices, no significant correlation was observed between salivary and serum TnI concentrations. This finding was consistent across correlation analyses, multivariable regression models, and longitudinal mixed-effects modelling. While earlier studies suggested moderate to strong associations between salivary and serum troponin concentrations [5,6], more recent evidence has questioned this relationship. In particular, Tran Hoa et al. [22] reported that salivary cardiac troponin concentrations do not consistently correlate with circulating serum concentrations, which is in agreement with the present findings. Although our findings are consistent with those reported by Tran Hoa et al. [22], who also observed no significant correlation between salivary and serum troponin concentrations, the present study extends previous knowledge in several important ways. First, we evaluated salivary TnI in a controlled model of perioperative myocardial injury following cardiac surgery, allowing assessment of biomarker dynamics under well-defined clinical conditions. Second, repeated measurements were obtained at multiple perioperative time points, enabling longitudinal evaluation of temporal changes. Third, several factors potentially influencing salivary biomarker measurements, including salivary flow rate, pH, hydration status, and renal function, were systematically assessed. Finally, measurements were performed using a high-sensitivity TnI assay following preliminary analytical verification in the salivary matrix. No significant associations between salivary and serum TnI concentrations were observed at any study time point. Similar findings were obtained in sensitivity analyses stratified according to surgical category. Although operative subgroups differed in underlying pathology and surgical characteristics, the consistency of findings across both CABG and valve surgery groups further strengthens the robustness of the primary study conclusion.

Collectively, available evidence suggests that salivary troponins are biologically detectable following myocardial injury, although their relationship with circulating concentrations remains inconsistent.

### 4.3. Potential Mechanisms Explaining the Lack of Correlation Between Salivary and Serum Troponin I

The absence of a significant association between salivary and serum TnI concentrations was the most important finding of the present study and warrants further consideration.

Several biological mechanisms may explain the absence of a direct relationship between salivary and serum TnI. Saliva is not simply an ultrafiltrate of plasma but rather a complex biological fluid generated through multiple secretory mechanisms [10,17,18]. The transfer of proteins from blood into saliva depends on molecular size, charge, glandular permeability, active transport processes, and local inflammatory conditions [10,17,18]. Given the relatively large molecular size of TnI, its passage from the circulation into saliva may be influenced by factors independent of circulating concentrations. In addition, circulating TnI may exist in different molecular forms, including intact protein and degradation fragments, which may differ in their ability to cross biological barriers and remain detectable within the salivary matrix. Consequently, salivary biomarker concentrations may exhibit kinetics that differ substantially from those observed in serum.

Additional mechanisms may further contribute to the observed dissociation between salivary and serum TnI concentrations. Recent evidence suggests that proteins may enter saliva through extracellular vesicles and exosome-mediated transport pathways rather than exclusively through passive diffusion [19,20]. Consequently, salivary biomarker concentrations may depend on local cellular processes within the salivary glands that are only partially related to circulating concentrations. Furthermore, salivary biomarker measurements may be influenced by proteolytic degradation within the oral cavity, as saliva contains numerous enzymes capable of modifying protein structure and immunoreactivity [12]. Perioperative physiological alterations associated with cardiac surgery, including systemic inflammation, vasoactive therapy, fluid shifts, and cardiopulmonary bypass, may additionally affect salivary gland permeability, secretion dynamics, and protein composition. Together, these mechanisms provide biologically plausible explanations for the absence of a significant association between salivary and serum TnI concentrations despite similar postoperative temporal trends observed in both biological matrices.

The finding that myocardial ischemia duration was associated with postoperative serum TnI concentrations but not with salivary TnI concentrations further supports the lack of a direct quantitative relationship between circulating and salivary troponin levels in the present study. Although salivary TnI concentrations increased postoperatively, they did not appear to reflect the extent of perioperative myocardial ischemic injury in the same manner as serum TnI. This observation is consistent with the absence of significant correlations between salivary and serum TnI concentrations and suggests that additional biological or analytical factors influence salivary troponin measurements.

A major strength of the present study is the extensive standardization of saliva collection and processing. Previous investigations have demonstrated that saliva collection methodology significantly influences biomarker measurements [11,12,13]. Salimetrics guidelines emphasize that collection method, handling procedures, and storage conditions may substantially affect sample quality and biomarker stability [13]. Granger et al. [12] highlighted the importance of standardized collection procedures in salivary biomarker research, while Chiu et al. [11] demonstrated significant differences among saliva collection techniques when evaluating biomarker detection. To minimize these sources of variability, saliva samples in the present study were collected using SalivaBio Oral Swab devices following pilot validation, thereby ensuring methodological consistency throughout the study.

Recent developments in saliva-based cardiovascular diagnostics have focused on improving analytical sensitivity through optimized sample preparation procedures. Westreich et al. [23] described a saliva-based point-of-care cardiac troponin I assay incorporating alpha-amylase depletion, demonstrating the feasibility of non-invasive troponin testing. Similarly, Franco-Martínez et al. [24] reported that filtration and alpha-amylase depletion significantly affect salivary biochemical measurements. These observations emphasize the importance of preanalytical sample preparation and may partly explain differences among published studies evaluating salivary troponins.

### 4.4. Influence of Pre-Analytical and Physiological Factors

Salivary flow rate represents another important source of variability in salivary diagnostics because increased secretion may theoretically dilute analyte concentrations [11,12]. However, no significant association between salivary flow rate and salivary TnI concentrations was observed in the present study. Furthermore, salivary flow rate was not associated with perioperative changes in salivary TnI concentrations. These findings suggest that dilution effects are unlikely to represent a major determinant of salivary TnI variability in cardiac surgical patients and support the robustness of the sampling protocol employed.

Salivary pH is another factor that may influence protein stability and immunoassay performance. Salivary pH was assessed using standardized pH indicator strips commonly employed for biological fluid analysis [25]. Although postoperative pH values decreased significantly, only weak associations were observed between pH and salivary TnI concentrations in the overall analyses. A subgroup analysis identified higher salivary troponin I concentrations among patients with lower salivary pH values; however, this finding emerged from an exploratory analysis and should therefore be interpreted cautiously. Given the absence of a significant overall association and the lack of adjustment for multiple testing, these observations should be considered hypothesis-generating rather than confirmatory. Nevertheless, they suggest that extreme alterations in salivary chemistry may influence troponin measurements and warrant further investigation in future studies.

The present study also evaluated the influence of renal function and hydration status on troponin concentrations. Reduced glomerular filtration rate was associated with higher serum TnI concentrations, consistent with previous evidence demonstrating elevated troponin concentrations in patients with impaired renal function. Positive fluid balance similarly influenced serum TnI concentrations and postoperative changes. In contrast, salivary TnI concentrations demonstrated limited susceptibility to these factors. This observation may represent a potential advantage of salivary biomarkers because serum troponin interpretation can be affected by several physiological and perioperative confounders.

Although renal function was associated with both serum and salivary TnI concentrations, additional sensitivity analyses stratified according to preoperative renal function suggested that differences in renal function were unlikely to be the primary explanation for the absence of a significant association between salivary and serum TnI concentrations. This finding supports the hypothesis that factors related to salivary transport mechanisms, local matrix characteristics, or assay performance may play a more important role than renal clearance in determining salivary troponin concentrations. However, larger studies including patients with a broader spectrum of renal dysfunction are required to confirm this observation.

### 4.5. Clinical Implications

Although salivary TnI cannot currently replace conventional blood-based troponin testing, the observed postoperative increase demonstrates that myocardial injury is associated with measurable changes in salivary troponin concentrations. These findings support continued investigation of saliva as a non-invasive diagnostic matrix and suggest that future applications may include serial monitoring, point-of-care testing, or screening approaches in situations where venous blood sampling is difficult or impractical. However, additional studies are required to clarify the biological mechanisms governing troponin transfer into saliva and to improve analytical methods before clinical implementation can be considered.

At present, however, the findings do not support clinical implementation of salivary troponin testing, and salivary TnI should be regarded as an investigational biomarker.

### 4.6. Strengths and Limitations

Several limitations should be acknowledged. First, this was a single-center study with a relatively modest sample size. Although the study was adequately powered for the predefined primary analyses, the sample size may have limited the ability to detect weaker associations and reduced the precision of subgroup analyses. Furthermore, the findings may not be fully generalizable to other cardiac surgical populations, institutions, or clinical settings. Although sensitivity analyses stratified by surgical category did not materially alter the principal findings, subgroup analyses were limited by the relatively small number of patients within individual procedure categories, particularly the combined or complex surgery subgroup.

Second, because almost all patients exceeded the predefined threshold for perioperative myocardial injury, only four participants remained below the diagnostic cutoff. Consequently, meaningful estimation of diagnostic accuracy metrics, including sensitivity, specificity, predictive values, and receiver operating characteristic (ROC) curve-derived area under the curve, was not possible. Therefore, the present investigation should be regarded as an exploratory biomarker study rather than a formal diagnostic accuracy study.

Third, the Access High-Sensitivity Troponin I assay was originally developed and validated for serum and plasma samples and has not undergone comprehensive analytical validation for use in saliva. Although preliminary analytical verification demonstrated acceptable repeatability and dilutional linearity, matrix-related analytical effects cannot be completely excluded. In addition, the evaluated concentration range was relatively narrow and represented only a limited portion of the assay measurement range. Consequently, it remains difficult to distinguish with certainty between biological mechanisms and potential analytical influences that may have contributed to the observed dissociation between salivary and serum TnI concentrations. Comprehensive analytical validation, including recovery studies, matrix-effect assessment, interference testing, limits of quantification, and stability studies, remains necessary before clinical application can be considered.

Fourth, although extensive efforts were undertaken to standardize saliva collection and processing, biological variability inherent to saliva cannot be completely eliminated. Additional sample-processing procedures, such as depletion of highly abundant salivary proteins, sample concentration techniques, or matrix-specific preparation protocols, were not applied. Such approaches may improve analytical sensitivity and should be explored in future studies.

Fifth, although the multivariable models included several clinically relevant covariates, residual confounding related to factors such as renal function, inflammatory response, transfusion requirements, and procedure-specific characteristics cannot be completely excluded. In addition, because multiple secondary and exploratory analyses were performed without formal adjustment for multiplicity, statistically significant findings from these analyses should be interpreted cautiously and require confirmation in independent cohorts.

The strengths of this study include its prospective longitudinal design, the use of a controlled model of myocardial injury, simultaneous collection of serum and saliva samples, and extensive standardization of saliva collection and processing procedures. In addition, the saliva collection method was preliminarily evaluated in a pilot study, supporting the selection of SalivaBio Oral Swab devices for standardized sampling throughout the study. Furthermore, major pre-analytical factors potentially affecting salivary biomarker measurements, including salivary flow rate, salivary pH, hydration status, and renal function, were systematically evaluated.

Despite these limitations, the study provides important evidence regarding the behavior of salivary TnI in a controlled perioperative setting. Although salivary TnI concentrations increased significantly following cardiac surgery, no significant independent association with serum TnI concentrations was observed after adjustment for relevant clinical factors. These findings demonstrate that measurable perioperative changes occur within the salivary compartment but do not support the use of salivary TnI as a surrogate marker of circulating cardiac troponin. At present, salivary TnI should therefore be regarded as an investigational biomarker. Future studies should focus on comprehensive analytical validation of high-sensitivity troponin assays in saliva, clarification of the biological mechanisms governing troponin transfer into saliva, and larger multicenter investigations to determine whether salivary troponin measurements provide clinically meaningful information beyond existing blood-based biomarkers.

## 5. Conclusions

Salivary TnI concentrations increased significantly following cardiac surgery, demonstrating that measurable perioperative changes occur within the salivary compartment. However, under the conditions investigated in this study, salivary TnI concentrations did not demonstrate a significant association with serum TnI concentrations and therefore cannot presently be considered a reliable surrogate marker for conventional blood-based troponin testing.

The biological significance of salivary troponin measurements remains incompletely understood and may be influenced by factors distinct from those affecting circulating troponin concentrations. Further analytical validation and clinical studies are required before definitive conclusions can be drawn.

Further studies are required to clarify the mechanisms of troponin transfer into saliva, establish comprehensive analytical validation of high-sensitivity troponin assays in the salivary matrix, and determine the potential clinical utility of salivary troponin measurements.

## Figures and Tables

**Table 1:** Baseline Demographic, Clinical, Laboratory, and Operative Characteristics of the Study Population (*n* = 54).

Characteristic	Value
Participants, *n*	54
**SEX, *n* (%)**	
Male	45 (83.3)
Female	9 (16.7)
**AGE GROUP, *n* (%)**	
<70 years	38 (70.4)
≥70 years	16 (29.6)
**ANTHROPOMETRIC CHARACTERISTICS**	
Age, years	65 (53.75–72.00)
Height, cm	175 (166.75–180.00)
Weight, kg	85.5 [77–96]
BMI, kg/m^2^	28.69 (26.28–30.72)
**NUTRITIONAL STATUS, *n* (%)**	
Normal weight	9 (16.7)
Overweight	27 (50.0)
Obesity class I	16 (29.6)
Obesity class II	2 (3.7)
**COMORBIDITIES, *n* (%)**	
Any comorbidity	44 (81.5)
Hypertension	40 (74.1)
Atrial fibrillation	6 (11.1)
Heart failure	1 (1.9)
Diabetes mellitus	16 (29.6)
Dyslipidemia	35 (64.8)
COPD	2 (3.7)
**SURGICAL PROCEDURE, *n* (%)**	
CABG	23 (42.6)
AVR	22 (40.7)
MVR	5 (9.3)
AVR + CABG	2 (3.8)
AVR + MVR	1 (1.9)
AVR + Ascending Aorta Procedure	1 (1.9)
**LABORATORY AND OPERATIVE CHARACTERISTICS**	
Preoperative GFR, mL/min/1.73 m^2^	84.3 (67.3–94.9)
Preoperative hemoglobin, g/L	132 [117–144]
Cardiopulmonary bypass time, min	76 [64–93]
Myocardial ischemia time, min	50 [37–61]

Data are presented as median (interquartile range) or *n* (%). Abbreviations: AVR, aortic valve replacement; CABG, coronary artery bypass grafting; MVR, mitral valve surgery; GFR, glomerular filtration rate; BMI, body mass index.

**Table 2:** Preliminary analytical verification of the Access High-Sensitivity Troponin I assay in saliva.

Parameter	Assessment	Result
Repeatability (high-concentration saliva sample)	10 consecutive measurements	Mean concentration 3.83 ng/L; CV = 3.1%
Repeatability (low-concentration saliva sample)	10 consecutive measurements	Mean concentration 1.14 ng/L; CV = 4.5%
Dilutional linearity	Serial mixtures of saliva samples with high and low troponin I concentrations	Progressive decrease in measured concentrations across dilution series
Linear regression analysis	Concentration versus dilution ratio	R^2^ = 0.98
Evaluated concentration range	Salivary troponin I	1.25–4.15 ng/L

Abbreviation: CV, coefficient of variation.

**Table 3:** Perioperative Laboratory Parameters, Hydration Status, Salivary Flow Rate, and Transfusion Requirements.

Variable	Preoperative	Postoperative (18–20 h)	Postoperative (42–44 h)	*p*-Value
LABORATORY PARAMETERS				
GFR (mL/min/1.73 m^2^)	84.7 (67.3–94.9)	79.9 (66.7–94.4)	85.3 (69.3–102.6)	0.02
CRP (mg/L)	1.7 (1.03–2.9)	48.7 (38.6–69.9)	115.2 (90.8–148.5)	<0.001
Leukocytes (×10^9^/L)	6.8 (5.6–8.0)	11.4 (9.2–13.4)	10.9 (9.1–12.6)	<0.001
Hemoglobin (g/L)	132 [117–144]	114 [101–120]	106 [97–112]	<0.001
SALIVARY FLOW RATE				
Salivary flow rate (mL/min)	1.05 (0.80–1.20)	0.60 (0.53–0.70)	0.80 (0.60–1.08)	<0.001
FLUID BALANCE AND TRANSFUSION REQUIREMENTS				
Fluid balance 0–24 h (mL)	—	−200 (−810 to 310)	—	—
Fluid balance 24–48 h (mL)	—	—	−320 (−1220 to 542.5)	—
Cumulative fluid balance (mL)	—	—	−490 (−1610 to 435)	—
Packed red blood cells (units) *	—	4 [2–10]	—	—
Fresh frozen plasma (units) **	—	3 [2–4]	—	—

Data are presented as median (interquartile range). * Number of transfused packed red blood cell units among patients receiving transfusion therapy (*n* = 37). ** Number of transfused fresh frozen plasma units among patients receiving transfusion therapy (*n* = 17). Abbreviations: GFR, glomerular filtration rate, CRP, creactive protein.

**Table 5:** Absolute and Relative Changes in Serum and Salivary Troponin I Concentrations and Their Associations with Clinical Variables.

Variable	18–20 h Postoperative	42–44 h Postoperative	*p*-Value
ABSOLUTE CHANGES			
Δ Serum TnI (ng/L)	2507.8 (1652.8–4719.2)	1159.3 (578.2–2290.6)	—
Δ Salivary TnI (ng/L)	4.75 (1.3–14.7)	3.55 (0.63–10.6)	—
RELATIVE CHANGES (%)			
%Δ Serum TnI	30,140.2 (13,935.8–55,938.4)	13,021.1 (6518.9–22,764.6)	—
%Δ Salivary TnI	138.7 (39.6–428.8)	105.8 (20.3–286.5)	—
SEX SUBGROUP ANALYSIS			
Female vs. male differences	No significant differences	No significant differences	all *p* > 0.05
AGE SUBGROUP ANALYSIS			
≥70 vs. <70 years differences	No significant differences	No significant differences	all *p* > 0.05
CORRELATIONS BETWEEN SERUM AND SALIVARY CHANGES			
Δ Serum TnI vs. Δ Salivary TnI (ρ)	0.106	0.033	0.47; 0.82
%Δ Serum TnI vs. %Δ Salivary TnI (ρ)	0.154	0.142	0.29; 0.34
CORRELATIONS WITH AGE AND ISCHEMIA TIME			
Age vs. Δ Serum TnI (ρ)	−0.172	0.164	>0.05
Age vs. Δ Salivary TnI (ρ)	0.146	0.251	>0.05
Ischemia time vs. Δ Serum TnI (ρ)	**0.351**	0.100	**0.01**
Ischemia time vs. Δ Salivary TnI (ρ)	0.088	−0.035	>0.05

Data are presented as median (interquartile range) or Spearman correlation coefficient (ρ). Detailed subgroup analyses according to sex and age are omitted from the main manuscript because no statistically significant differences were observed. Bold values indicate statistically significant associations (*p* < 0.05). Abbreviations: TnI, troponin I; Δ, absolute change; %Δ, relative change.

**Table 6:** Association between Salivary pH and Salivary Troponin I Concentrations.

Variable	Preoperative	Postoperative (18–20 h)	Postoperative (42–44 h)	*p*-Value
SALIVARY pH				
pH value	7.0 (6.8–7.3)	6.1 (6.1–6.4)	6.4 (6.1–6.9)	<0.001
CORRELATION WITH SALIVARY TROPONIN I (ρ)				
Salivary TnI vs. pH	0.009	−0.168	−0.203	>0.05
Δ Salivary TnI vs. pH	−0.113	−0.123	−0.144	>0.05
LINEAR REGRESSION ANALYSIS				
β coefficient	−0.136	−0.257	−0.474	0.59; 0.40; 0.07
R^2^	0.008	0.016	0.073	—
pH SUBGROUP ANALYSIS (<6 vs. ≥6)				
Salivary TnI (ng/L)	—	13.9 vs. 9.0	**14.1 vs. 5.8**	**0.02**
Δ Salivary TnI (ng/L)	—	8.8 vs. 4.4	**10.9 vs. 2.7**	**0.02**

Data are presented as median (interquartile range), Spearman correlation coefficient (ρ), or regression coefficient (β). Salivary pH values were compared using Friedman repeated-measures analysis with Conover post hoc testing. Bold values indicate statistically significant associations (*p* < 0.05). Abbreviations: TnI, troponin I; Δ, absolute change; β, standardized regression coefficient.

**Table 7:** Summary of the Effects of Renal Function, Hydration Status, and Salivary Flow on Troponin I Concentrations.

Factor	Outcome Variable	Association (ρ)	*p*-Value
Perioperative GFR changes	GFR over time	–	0.02
Preoperative GFR	Salivary TnI (preoperative)	**0.373**	**0.007**
Preoperative GFR	Salivary TnI (18–20 h)	**0.305**	**0.030**
Preoperative GFR	Salivary TnI (42–44 h)	**0.314**	**0.020**
Postoperative GFR	Salivary TnI	NS	>0.05
Preoperative GFR	Serum TnI (preoperative)	**−0.343**	**0.020**
Preoperative GFR	Serum TnI (18–20 h)	**−0.503**	**<0.001**
Preoperative GFR	Serum TnI (42–44 h)	**−0.321**	**0.020**
Postoperative GFR (42–44 h)	Serum TnI (preoperative)	**−0.286**	**0.040**
Postoperative GFR (42–44 h)	Serum TnI (18–20 h)	**−0.344**	**0.010**
GFR	Absolute change in serum TnI (Δserum TnI)	NS	>0.05
GFR	Absolute change in salivary TnI (Δsalivary TnI)	NS	>0.05
Fluid balance (0–24 h)	Serum TnI (42–44 h)	**0.335**	**0.020**
Fluid balance (0–24 h)	Salivary TnI	NS	>0.05
Fluid balance (0–24 h)	ΔSerum TnI (18–20 h)	**0.282**	**0.040**
Fluid balance (0–24 h)	ΔSerum TnI (42–44 h)	**0.342**	**0.020**
Cumulative fluid balance	ΔSerum TnI (42–44 h)	**0.411**	**0.010**
Fluid balance	ΔSalivary TnI	NS	>0.05
Fluid balance	Relative changes in serum TnI	NS	>0.05
Fluid balance	Relative changes in salivary TnI	NS	>0.05
Salivary flow rate	Salivary TnI concentrations	NS	>0.05
Salivary flow rate	Absolute changes in salivary TnI	NS	>0.05
Salivary flow rate	Relative changes in salivary TnI	NS	>0.05
Categories of cumulative fluid balance	Serum TnI, salivary TnI, pH, absolute and relative TnI changes	NS	>0.05

Abbreviations: GFR = glomerular filtration rate; TnI = troponin I; NS = not significant; Δ = absolute change from baseline.

**Table 8:** Multivariable Linear Regression Models of Independent Predictors of Postoperative Serum Troponin I Concentrations.

Model	Predictor	β	95% CI for β	*p*-Value
Model I	ln Salivary TnI (24 h)	0.09	−0.24 to 0.41	0.60
	Age	−0.01	−0.04 to 0.02	0.53
	Sex	0.06	−0.66 to 0.78	0.86
	Ischemic time	0.01	−0.003 to 0.03	0.13
	Fluid balance (0–24 h)	**0.001**	**0.001 to 0.002**	**0.008**
	Model statistics	R = 0.476; R^2^ = 0.227; Adjusted R^2^ = 0.141; F(5, 45) = 2.64		**0.04**
Model II	ln Salivary TnI (48 h)	0.02	−0.01 to 0.05	0.12
	Age	−0.73	−1.54 to 0.07	0.07
	Sex	0.01	−0.01 to 0.02	0.30
	Ischemic time	−0.20	−0.57 to 0.17	0.29
	Cumulative fluid balance	0.00	0.00 to 0.00	0.13
	Model statistics	R = 0.448; R^2^ = 0.201; Adjusted R^2^ = 0.080; F(5, 33) = 1.70		0.17

Abbreviations: TnI = troponin I; CI = confidence interval; β = regression coefficient. Bold values indicate statistical significance (*p* < 0.05).

**Table 9:** Linear Mixed-Effects Model of the Association Between Salivary and Serum Troponin I During the Perioperative Period.

Predictor	β (95% CI)	*p*-Value
Ln (Salivary TnI)	0.08 (−0.12 to 0.29)	0.43
Time: 18–20 h postoperatively	**5.57 (5.22 to 5.92)**	**<0.001**
Time: 42–44 h postoperatively	**4.80 (4.47 to 5.12)**	**<0.001**

Model: Linear mixed-effects model with patient-specific random intercept; dependent variable: ln(serum TnI); 54 patients, 156 observations; reference category: preoperative measurement. Abbreviations: TnI = troponin I; CI = confidence interval; β = regression coefficient.

## Data Availability

The original contributions presented in this study are included in the article/Supplementary Material. Further inquiries can be directed to the corresponding author.

## Supplementary Materials

The following supporting information can be downloaded at: https://www.mdpi.com/article/10.3390/diagnostics16132077/s1. Individual salivary hs-cTnI trajectories are presented in Supplementary Figure S1, illustrating the marked inter-individual variability in perioperative salivary troponin responses.

## Abbreviations

AVR	Aortic Valve Replacement
BMI	Body Mass Indeks
CABG	Coronary Artery Bypass Grafting
CI	Confidence Interval
CK-MB	Creatine Kinase-MB
CLIA	Chemiluminescent Immunoassay
COPD	Chronic Obstructive Pulmonary Disease
CRP	C-Reactive Protein
TnI	Cardiac Troponin I
eGFR	Estimated Glomerular Filtration Rate
ESC	European Society of Cardiology
GFR	Glomerular Filtration Rate
IQR	Interquartile Range
ln	Natural Logarithm
MI	Myocardial Infarction
MVR	Mitral Valve Replacement/Repair
ROC	Receiver Operating Characteristic
SD	Standard Deviation
SOS	SalivaBio Oral Swab
TnC	Troponin C
TnI	Troponin I
TnT	Troponin T
β	Regression Coefficient
ρ	Spearman Correlation Coefficient
