# Supplementary figures and images for: Relationship Between Salivary and Serum Cardiac Troponin I in Patients Undergoing Cardiac Surgery: A Prospective Longitudinal Observational Study
Source: Diagnostics (Basel). 2026 Jul 2;16(13):2077. doi: 10.3390/diagnostics16132077 (PMC13361651; doi:10.3390/diagnostics16132077)

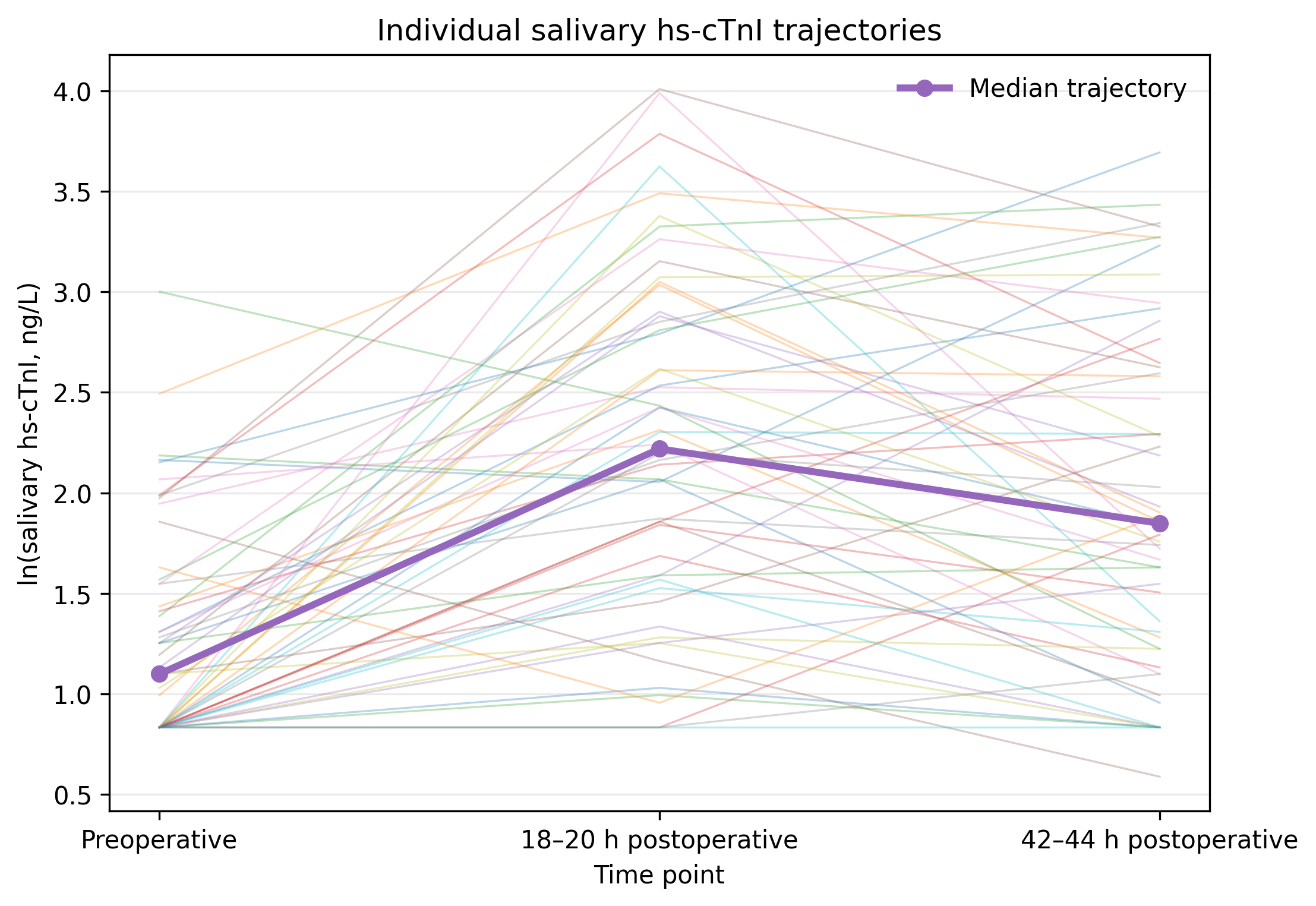

Supplement: Supplementary file 1 [file diagnostics-16-02077-s001.zip › diagnostics-4380697-supplementary.png]
